# A 12-Week Strict Low FODMAP Diet Reduces the Severity Levels of Fatigue, Depression, Anxiety, and Inattention in Patients with Irritable Bowel Syndrome

**DOI:** 10.1016/j.cdnut.2025.107483

**Published:** 2025-06-06

**Authors:** Sol Maja G Bjørkevoll, Eline M Randulff Hillestad, Gülen A Lied, Erica S Teige, Elisabeth K Steinsvik, Birgitte Berentsen, Astri J Lundervold

**Affiliations:** 1 Department of Pediatric and Adolescent Medicine, Innlandet Hospital Trust, Lillehammer, Norway; 2 Department of Global Public Health and Primary Care, Centre for International Health, University of Bergen, Bergen, Norway; 3 Center for Nutrition, Department of Clinical Medicine, Faculty of Medicine, University of Bergen, Bergen, Norway; 4 Division of Gastroenterology, Department of Medicine, National Center for Functional Gastrointestinal Disorders, Haukeland University Hospital, Bergen, Norway; 5 Department of Clinical Medicine, Faculty of Medicine, University of Bergen, Bergen, Norway; 6 Department of Biological and Medical Psychology, University of Bergen, Bergen, Norway

**Keywords:** irritable bowel syndrome, FODMAP, gut–brain axis, fatigue, depression, attention

## Abstract

**Background:**

The low FODMAP diet (LFD) has been shown to alleviate gastrointestinal symptoms in patients with irritable bowel syndrome (IBS). However, the impact of the LFD on coexisting symptoms of fatigue, anxiety, depression, and cognitive functions remains unclear.

**Objectives:**

This study aims to investigate the effects of a 12-wk strict LFD on symptoms of fatigue, depression, anxiety, and inattention in patients with moderate-to-severe IBS-D (diarrhea-predominant), and IBS-M (mixed diarrhea and constipation).

**Methods:**

Participants with IBS were included in an open-label, single-center, 12-wk dietary intervention conducted at Haukeland University Hospital in Bergen, Norway. They followed a strict LFD guided by a registered dietitian and completed questionnaires assessing fatigue [Chalder Fatigue Scale (CFQ-11)] and anxiety and depression [Hospital Anxiety and Depression Scale (HADS)] and performed a test of attention [the third edition of the Connors’ continuous performance test (CPT-3)] at baseline and 12-wk follow-up. Data were analyzed using Wilcoxon signed-rank tests to evaluate changes from baseline to post intervention.

**Results:**

Thirty-six participants completed the intervention. Statistically significant reductions were observed in symptoms of fatigue (CFQ-11, *P <* 0.013) and symptoms of anxiety (HADS-A, *P <* 0.006). Most of the patients were defined as noncases of fatigue and anxiety following the intervention. Performance on the CPT-3 revealed improvements in measures of inattention.

**Conclusions:**

A 12-wk strict LFD intervention significantly alleviated coexisting symptoms of fatigue, anxiety, and depression, and improved performance on a psychometric test of attention. These findings suggest that dietary management plays a crucial role in improving both physical and mental health in patients with IBS.

This study was registered at clinical trial as NCT04296552 (https://clinicaltrials.gov/study/NCT04296552?term=NCT04296552&rank=1).

## Introduction

Irritable bowel syndrome (IBS) is a common gastrointestinal (GI) disorder affecting up to 15% of the population worldwide [1]. It is characterized by recurrent abdominal pain associated with defecation or altered bowel habits, and is classified into subtypes based on predominant stool form according to the Bristol stool form scale [2]: Diarrhea-predominant (IBS-D), constipation-predominant (IBS-C), or mixed bowel habits (IBS-M). This classification helps tailor management strategies to the specific needs of subgroups of patients, yet the underlying causes of IBS remain elusive.

The pathophysiology of IBS is complex and multifactorial, involving disturbances in the gut–brain axis (GBA) [3]. The GBA is a bidirectional communication network that includes the enteric and central nervous system and communication throughout neural, immunological, and endocrine pathways [4]. Disturbances in this axis are thought to contribute not only to the GI symptoms but also to a range of commonly reported coexisting symptoms associated with psychological distress [[5], [6], [7]].

IBS and its coexisting symptoms significantly affect daily life, including work, productivity, social interactions, and eating habits [[8], [9], [10]]. Studies have consistently shown that patients with IBS have a significantly reduced quality of life [[11], [12], [13]]. Furthermore, patients with IBS with coexisting symptoms and disorders tend to report more severe IBS symptoms and even lower quality of life than patients with IBS with symptoms restricted to core GI symptoms [14]. Despite the considerable burden of IBS, treatment options are limited, and many patients do not achieve satisfactory symptom control with standard therapies.

One well-documented and effective dietary therapy for managing IBS is the low FODMAP (fermentable oligosaccharides, disaccharides, monosaccharides, and polyols) diet (LFD) [[15], [16], [17]]. The LFD significantly improves core GI symptoms, particularly in patients with IBS-D and IBS-M, with symptom relief reported in 50%–80% [18]. The diet works by reducing the intake of short-chain carbohydrates that are poorly absorbed in the small intestine, thereby decreasing fermentation, gas production, and excess water increase in the gut, which can alleviate bloating, pain, and altered bowel habits.

However, the impact of the LFD on extraintestinal symptoms such as fatigue, anxiety, and depression remains unclear. Although some studies have reported improvements in these symptoms following the LFD [19,20], others, including a meta-analysis, found no significant effect [21]. Moreover, prior research has primarily focused on short-term interventions (typically 4–6 wk). Importantly, to our knowledge, no study has investigated the effect of LFD on cognitive function.

Previous findings from our research group have demonstrated that participants with IBS, at a group level, exhibit severe symptoms of fatigue and emotional problems [5,22] as well as impairments in memory, attention, and executive function on psychometric tests [23,24]. Additionally, it has been shown that a strict 12-wk LFD led to a significant reduction in intestinal IBS symptoms [13]. Building on these findings, this study aims to assess changes in symptoms of fatigue, depression, anxiety, and attention-related performance from baseline to the end of this 12-wk dietary intervention. By using a longer intervention period than typical LFD studies and measuring emotional and cognitive domains in addition to GI symptoms, this study fills an important research gap and contributes new insight into the broader effects of dietary treatment in IBS.

## Methods

### Study procedures and participants

The Bergen Brain Gut (BBG) study is an open-label, single-center, 12-wk dietary intervention for patients with moderate or severe IBS-D and IBS-M. The study was conducted at Haukeland University Hospital in Bergen, Norway, from May 2019 to June 2021, with inclusion and exclusion criteria (Table 1) [25]. Organic diseases were ruled out via general blood samples, upper endoscopy, sigmoidoscopy, and abdominal ultrasound, and the diagnosis was confirmed by a gastroenterologist. All participants provided written consent and the project was conducted following the ethical requirements of the Declaration of Helsinki. The study protocol of the BBG study is available elsewhere [25]. Power calculations were performed before study initiation and are described in the study protocol [25]. The BBG study was approved by the Southeast Regional Ethical Committees for medical and health research ethics in Norway (REK2015-01621).

**TABLE 1:** Inclusion and exclusion criteria for the participants.

Inclusion criteria	• 18–65 y/o • Fulfill Rome IV criteria and IBS-SSS > 175 • Normal diet ≥3 wk before inclusion
Exclusion criteria	• Low FODMAP diet or probiotics in the last 3 wk
	• Not able to participate due to psychological factors
	• Antibiotics during the last 3 mo
	• Pregnant or PCOS
	• Vegetarian or vegan
	• Permanent medication use
	• Traveled outside Europe in the last 3 wk
	• Previous intestinal surgery, except appendectomy
	• Organic disease (CD, IBS, endometriosis, diabetes, *Helicobacter pylori* infection, neurological diseases except migraine)
	• Alarm symptoms (anemia, onset of IBS after 45 y/o nocturnal symptoms, blood in stool, family history of colorectal cancer, fever in association with diarrhea)

Abbreviations: CD, celiac disease; DGBI, disorders of the gut–brain interaction; FODMAP, fermentable, oligo-, di-, monosaccharides and polyols; GI, gastrointestinal; HC, healthy controls; IBD, inflammatory bowel disease; IBS, irritable bowel syndrome; IBS-SSS; irritable bowel syndrome – symptom severity score; PCOS, polycystic ovarian syndrome; y/o, years old.

Participants followed a 12-wk strict LFD, guided by a registered dietitian. The procedure included 3 in-person consultations (baseline and weeks 4 and 12) and 1 phone consultation at week 8. The registered dietitian was available via phone and e-mail throughout the entire intervention period. Patients received instructions on FODMAP reintroduction after the completion of the 12-wk study period. Dietary adherence was assessed by calculating FODMAP intake from 3-d dietary records at baseline and week 12, as detailed in Hillestad et al. [13].

### Questionnaires and the psychometric test

At baseline and postintervention, the patients completed standardized questionnaires assessing symptoms of fatigue, anxiety, and depression and performed a computerized test of attention.

The Chalder Fatigue Scale (CFQ-11) was included to detect clinical cases of fatigue, assess the severity of fatigue symptoms, and track changes in severity level over time [26]. It contains 11 questions that measure physical (questions 1–7) and mental fatigue (questions 8–11). The bimodal scoring system assigns 1 point if a symptom is reported as more than usual or much more than usual, and 0 points otherwise. The global fatigue score ranges from 0 to 11, where a score ≥4 indicates a fatigue case, and ≤3 defines noncases [26].

Hospital Anxiety and Depression Scale (HADS) is a questionnaire designed to screen for and measure symptoms of anxiety and depression in adults [27], based on 14 questions, divided into an anxiety (HADS-A) and depression (HADS-D) subscale (7 items each, max score = 21). A total HADS score (HADS-T) ranges from 0 to 42 [27] and is used as a measure of symptom severity. On the basis of subscale scores, participants were categorized as noncase (<8), doubtful case (8–11), or case (>11) [27].

Continuous performance test-3 (CPT-3) is a computerized test of attention designed to assess inattentiveness, impulsivity, sustained attention and vigilance in individuals [28]. The test lasts 14 min, and presents participants with a series of letters appearing on the screen, requiring them to press the space bar for every letter except “X.” The interstimulus interval, that is, the time between the presentation of 2 letters, varies to assess attention under different conditions [28]. See Table 2 for a description of the test variables.

**TABLE 2:** Overview of the measures included in the CPT-3 test [26].

Measures	Description
Detectability	The ability to identify targets as distinct from nontargets
Omissions	The number of missed target responses
Commissions	Occurrences of responses to nontarget stimuli that are incorrect
Perseverations	Responses that occur within 100 ms following a previous response
HRT	The mean response time for correct responses
HRT SD	The variability of response times for correct responses
Variability	The consistency of the response times
HRT block change	Changes in reaction time across different blocks
HRT ISI change	Changes in reaction time across different interstimulus intervals

Abbreviations: CPT-3, continuous performance test-3; HRT, hit reaction time; IBS; irritable bowel syndrome; ISI, interstimulus interval.

IBS–Symptom Severity Scale (IBS-SSS) was used to assess GI symptom severity at baseline and week 12. We have previously reported a significant reduction in IBS-SSS score in this cohort following the 12-wk strict LFD [13]. In this study, IBS-SSS change scores were used to investigate its association with changes in symptoms of fatigue, anxiety, depression, and inattention.

### Statistics

Continuous variables were reported as means and SDs or medians and IQR, depending on the data distribution. Categorical variables were reported as frequencies and percentages. To assess within-group differences from baseline to post intervention, Wilcoxon signed-rank tests were performed. Bonferroni-adjusted α levels were applied to control for multiple comparisons. To quantify effect sizes, Cohen’s *d* values were calculated, with cutoffs of 0.20 (small), 0.50 (medium), and 0.80 (large). Analyses based on change scores were conducted on a reduced sample due to missing responses at either baseline or week 12. To explore whether changes in core GI symptoms were associated with changes in other symptom domains, Spearman correlations were conducted between the change in IBS-SSS scores and changes in fatigue, anxiety, depression, and attention measures. Because the study lacked a control group, statistical analyses are limited to within-group comparisons and causal conclusions cannot be drawn. Statistical analyses were conducted in R (version 4.41; R Core Team, 2024), including the effsize package for effect size calculations. Figures were generated using JASP (Version 0.18.3).

## Results

**FIGURE 1:**
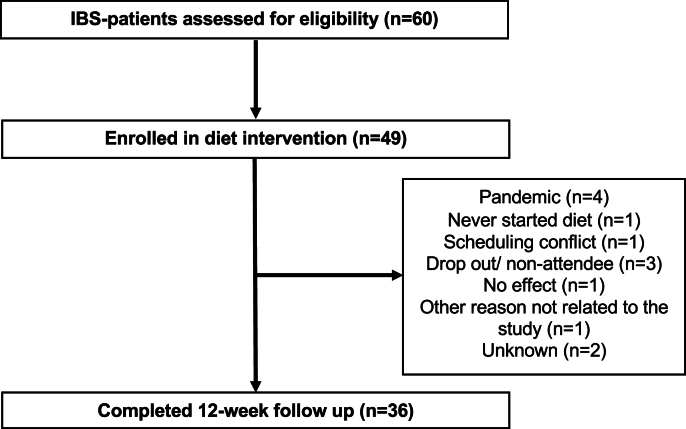
Flow chart of the diet intervention. IBS, irritable bowel syndrome.

Of 60 participants with IBS assessed for the dietary intervention, 49 were enrolled in the 12-wk strict LFD. Thirteen (27%) participants did not complete the intervention, resulting in 36 patients with IBS who performed the 12-wk LFD (Figure 1). Most of the participants were female, and all had moderate or severe IBS at baseline. Baseline characteristics for those who completed the intervention are summarized in Table 3. Compared with completers, noncompleters were more often females (92% compared with 67%) and were less educated. No notable difference was observed in age, social status, or IBS subtype (Supplemental Table 1).

**TABLE 3:** Baseline characteristics of the participants (*n* = 36)^1^.

	*n*	Values
Diagnoses		
IBS-D	15	42
IBS-M	21	58
Demographics		
Age	36	37 (11)
Female	24	67
Social status		
Single	9	25
Cohabitant/ married	27	75
Education (y)		
<13	8	22
13−17	13	36
>17	15	42
IBS symptom severity		
Moderate IBS	27	75
Severe IBS	9	25

Abbreviations: IBS, irritable bowel syndrome.

1 All values are given in percentages, except age which is given in mean (SD).

**FIGURE 2:**
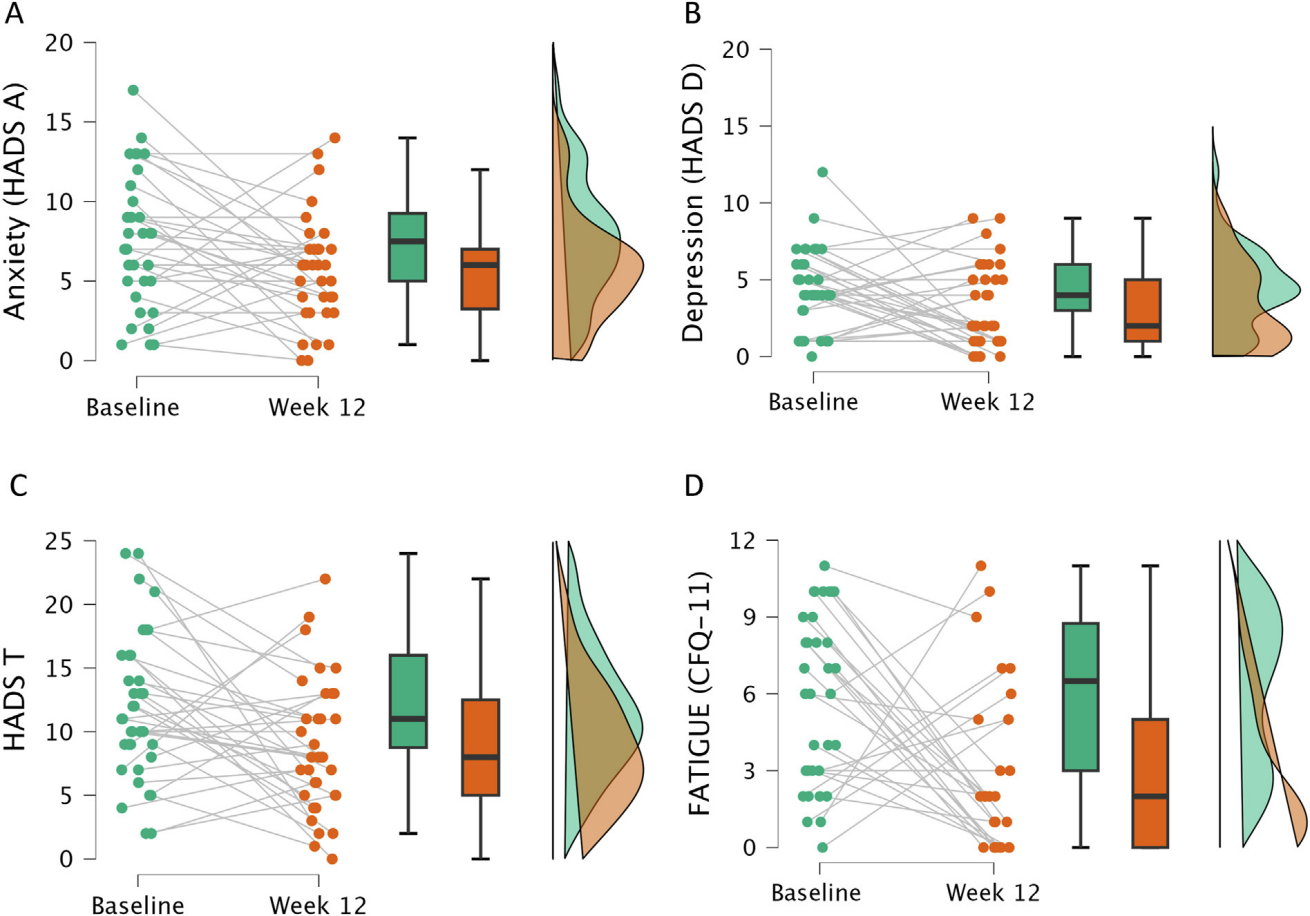
Individual and median changes in self-reported symptoms of anxiety (A), depression (B), combined anxiety and depression (C), and fatigue (D) from baseline to week 12. CFQ-11, Chalder Fatigue Scale; HADS-T, Hospital Anxiety and Depression Total Score; HADS-D, Hospital Anxiety and Depression Scale Depression Subscale; HADS-A, Hospital Anxiety and Depression Scale Anxiety Subscale.

Following the 12-wk strict LFD, there was a significant reduction in self-reported fatigue (*P* < 0.013, *d* = 0.8) (Figure 2D and Table 4). At baseline, 68% of the participants were classified as having fatigue, decreasing to 32% post intervention. Additionally, the reduction was found on each of the CFQ-11 from baseline to the follow-up at week 12 (Supplemental Table 2). At both time-points, physical fatigue scores (questions 1–7) tended to be higher than mental fatigue scores (questions 8–11).

**TABLE 4:** Changes in measures of anxiety, depression, fatigue, and attention from baseline to the 12-wk follow-up after the dietary intervention^1^.

	Baseline^2^	Follow-up^3^	Diff^4^	*P* (exact)^5^	Cohen’s *d*
Fatigue					
CFQ11	6.5 (3.0, 8.8)	2.0 (0.0, 5.0)	2.0 (0.0, 5.0)	<0.013^6^	0.816∗∗
Anxiety & depression					
HADS-T	11.0 (8.8, 16.0)	8.0 (5.0, 12.5)	3.0 (1.0, 5.0)	<0.013^6^	0.524∗
HADS-A	7.5 (5.0, 9.2)	6.0 (3.2, 7.0)	2.0 (−0.8, 3.0)	<0.013^6^	0.463
HADS-D	4.0 (3.0, 6.0)	2.0 (1.0, 5.0)	1.0 (−0.8, 3.0)	0.018	0.426

Cohen’s *d*/effect size: “∗” indicates a medium effect size (Cohen’s d values ranging from 0.5 to 0.8) and “∗∗” indicates a large effect size (Cohen’s *d* values larger than 0.8).

Abbreviations: CFQ-11, Chalder Fatigue Scale; HADS-T, Hospital Anxiety and Depression Scale Total Score; HADS-D, HADS Depression Subscale; HADS-A, HADS Anxiety Subscale.

1 Median and IQRs are presented for the scores at baseline and follow-up, and the *Diff* is the median difference between the 2 time-points.

2 *n* = 34 for fatigue, *n* = 36 for anxiety and depression.

3 *n* = 28 for fatigue, *n* = 34 for anxiety and depression.

4 *n* = 27 for fatigue, *n* = 34 for anxiety and depression.

5 Wilcoxon signed-rank tests.

6 *P* values are considered statistically significant after Bonferroni correction (*P <* 0.05/4).

Participants also reported a reduction in self-reported symptoms of anxiety (*P* < 0.013) and depression (*P* = 0.018), both with medium effect sizes. These *P* values were below or close to the α after Bonferroni correction (*P* = 0.013) (Figure 2A–C and Table 3). At baseline, 50% of the participants were defined as doubtful cases or cases of anxiety. Following the intervention, the number of doubtful cases and cases of anxiety decreased to 21%.

Participants demonstrated improved performance on the CPT-3 test from baseline to the 12 wk follow-up (Table 5). Significant improvements were observed in 3 critical domains—detectability, variability, and hit reaction time-SD—with statistically significant *P* values even after Bonferroni correction (<0.006*)*. The effect sizes for the last 2 were substantial, indicating a moderate-to-large effect of the intervention on these parameters. Marginally significant improvements were also noted in the Omissions and Commissions scores, though these did not meet the threshold for statistical significance after correction for multiple comparisons. These results collectively show improved attentional control, as the patients show more consistent, accurate, and stable performance across the assessment—all hallmarks of reduced inattention.

**TABLE 5:** Changes in measures of attention from baseline to the 12-wk follow-up after the dietary intervention.^1^

	Baseline (*n* = 36)	Follow-up (*n* = 31)	Diff (*n* = 31)	*P* (exact)^2^	Cohen’s *d*
CPT-3					
Detectability	46.5 (42.0, 56.0)	43.0 (39.5, 49.0)	3.0 (0.0, 6.5)	<0.006^3^	0.457
Omissions	45.0 (45.0, 47.2)	45.0 (44.0, 45.5)	0.0 (0.0, 2.0)	0.014	0.498
Commissions	48.5 (42.8, 57.5)	44.0 (41.0, 56.0)	2.0 (0.0, 5.5)	0.013	0.216
Perseverations	46.0 (45.0, 48.0)	46.0 (45.0, 48.0)	0.0 (0.0, 0.0)	0.670	0.178
HRT	46.5 (44.0, 52.2)	48.0 (42.5, 51.0)	1.0 (−3.5, 3.5)	0.618	0.071
HRT SD	44.5 (39.8, 50.2)	41.0 (37.5, 44.0)	3.0 (2.0, 6.0)	<0.006^3^	0.660∗
Variability	45.0 (43.0, 48.0)	44.0 (39.5, 46.0)	2.0 (0.0, 5.0)	<0.006^3^	0.659∗
HRT block change	49.0 (43.8, 53.2)	47.0 (42.0, 51.0)	2.0 (−4.5, 6.5)	0.576	0.078
HRT ISI change	48.5 (42.8, 52.2)	48.0 (46.5, 52.0)	1.0 (−4.0, 2.0)	0.680	−0.050

Cohen’s *d*/effect size: “∗” indicates a medium effect size (Cohen’s *d* values ranging from 0.5 to 0.8).

Abbreviations: CPT-3, Continuous Performance Test-3; HRT, hit reaction time; IBS; irritable bowel syndrome; ISI, interstimulus interval.

1 Median and IQRs for the scores at baseline and follow-up, and the median difference (*Diff*) between the 2 time-points.

2 Wilcoxon signed-rank tests.

3 *P* values are considered statistically significant after Bonferroni correction (*P <* 0.05/9).

Associations between changes in core GI symptoms measured by IBS-SSS and changes in fatigue, anxiety, depression, and attention were examined using Spearman correlation. No statistically significant associations were observed (Supplemental Table 3).

## Discussion

This study showed significant clinical improvement in patients with IBS from pre- to post–12-wk LFD intervention. Specifically, the participants exhibited a reduction in symptoms of fatigue, anxiety, and depression, alongside enhanced performance on a computerized test of attention. The observed multidomain improvement supports a system view on IBS that extends beyond GI symptoms [29] and underscores the potential therapeutic value of nutritional interventions for addressing the cognitive and affective components of this disorder.

The significant reduction in both mental and physical fatigue aligns with findings from Kortlever et al. [19] and Eswaran et al. [20]. Kortlever et al. [19] followed 111 participants through a 6-wk LFD and observed significant reductions in symptoms of fatigue, both after the restriction phase and 20 wk after the FODMAP reintroduction phase. Similarly, Eswaran et al. [20] found a significant decrease in fatigue scores among their 92 participants after a 4-wk LFD. Our study implemented a 12-wk restriction phase, which is longer than in most previous studies. The results demonstrated a large effect size in reducing the severity of fatigue symptoms, with fewer participants meeting the criteria as “fatigue cases” after the intervention.

These findings strongly indicate that an LFD may be an effective approach for treating fatigue in patients with IBS.

We also observed a reduction in symptoms of anxiety and depression. Several studies have reported similar reductions in anxiety and depression following 4–6 wk of LFD [19,20,30]. However, Schumann et al. [31], who conducted an LFD intervention for the same duration as the current study (12 wk), reported a significant decrease in depression symptoms but no effect on anxiety. In contrast, Pedersen et al. [32] found no significant changes in anxiety or depression after 6 wk of LFD. Like other studies, we found a mean/median reduction of 1−2 points in each of the HADS-A (anxiety) and HADS-D (depression) subscales. Although these changes reached statistical significance in our study, their clinical relevance may be limited in that the HADS subscales range from 0 to 21. A reduction of 1–2 points thus represents only ∼5%–10% of the total possible score. Nevertheless, our findings make an important contribution by demonstrating that extended adherence to an LFD (12-wk) does not negatively impact emotional well-being. This is particularly relevant considering concerns about the potential negative psychological effects of restrictive diets on mental health. Our data suggest that patients even experienced improvements.

The observed improvements in CPT-3 performance further support the impact of the LFD. To our knowledge, this is the first study showing improved performance on measures from a computerized test of attention after an LFD intervention. Although cognitive impairments have been reported in IBS [23,24], they remain largely overlooked in treatment studies. Combined with improvement on the subscale of cognitive fatigue and symptoms of anxiety and depression, our findings suggest that dietary management with LFD may both directly and indirectly affect cognitive health, and this further emphasizes the importance of interconnections between the gut and brain [33].

Exploratory correlation analyses did not reveal significant associations between improvements in GI symptoms and changes in symptoms of fatigue, anxiety, depression, or inattention. Although several symptom domains improved at the group level, the lack of significant correlations suggests that these improvements may reflect distinct response patterns rather than a unified effect across individuals. These preliminary findings highlight the complexity of symptom trajectories and warrant further investigation in larger, controlled studies.

The current study explored the effects on emotional and cognitive function of a 12-wk strict LFD intervention guided by a registered dietitian and thus represents an area of research that is largely unexplored. To our knowledge, this is the first study to investigate the impact of the LFD on a broad range of emotional domains and attention. We also believe that the 12-wk duration of the intervention was important to detect changes assumed to be related to the brain–gut axis. This extended duration further allowed us to observe the potential extended effects of the LFD’s restriction phase.

The involvement of a registered dietitian in providing guidance throughout the intervention is another strength of this study. This professional support increased the probability of high compliance and ensured dietary adequacy among participants. As shown in a previous publication [13], we achieved high compliance with the LFD intervention shown in a low intake of total FODMAPs at week 12. Additionally, our sample of participants reflected the general gender and age distribution of patients with IBS [[34], [35], [36]], increasing the external validity of our findings.

Despite its promising findings, this study has several limitations. The open-label design without a control group introduces a potential bias, as both participants and researchers were aware of the intervention. This raises the possibility of a placebo effect contributing to symptom improvement. Additionally, the sample size was relatively small, which may have reduced the statistical power. Although initial power calculations suggested that a sample size of 60 would be adequate [25], the COVID-19 pandemic resulted in unforeseen recruitment challenges and participant drop-out, which impacted the final number of participants. Although our findings are promising, this study should be interpreted as a pilot study. The results should be confirmed in larger, controlled trials.

In conclusion, this study suggests that a 12-wk strict LFD significantly alleviates fatigue, anxiety, and depression, and improves performance on a test of attention in patients with moderate-to-severe IBS-D and IBS-M. These findings highlight the potential of dietary interventions as part of a holistic approach to IBS management.

In summary, a strict LFD improved extraintestinal symptoms in patients with IBS, including fatigue, emotional distress, and attention, suggesting multidimensional effects beyond GI symptom relief.

## Author contributions

The authors’ responsibilities were as follows – SMGB, BB, AJL: designed the study; SMGB: performed the statistical analysis; SMGB, AJL: wrote the manuscript; and all authors: collected the data, critically reviewed, and approved the final manuscript.

## Data availability

The data and code that support the findings of this study are available when accepted for publication (https://github.com/astrijl/lowFODMAP)

## Funding

This research was funded by the Research Council of Norway (grant ID FRIMED-BIO276010) and Helse Vest’s Research Funding (grant ID HV912243) and by the Trond Mohn Research Foundation, grant number BFS2018TMT0, and from The Research Council of Norway, project number 294594.

## Conflict of interest

The authors report no conflicts of interest.
