# Supplementary material for: A 12-Week Strict Low FODMAP Diet Reduces the Severity Levels of Fatigue, Depression, Anxiety, and Inattention in Patients with Irritable Bowel Syndrome
Source: Curr Dev Nutr. 2025 Jun 6;9(7):107483. doi: 10.1016/j.cdnut.2025.107483 (PMC12246592; doi:10.1016/j.cdnut.2025.107483)
Supplement: Multimedia component 1 [file mmc1.docx]

**A 12-week strict low-FODMAP diet reduced the severity levels of fatigue, depression, anxiety, and inattention in patients with irritable bowel syndrome**

First author: Sol Maja G Bjørkevoll

Supplementary Table 1: Baseline characteristics among completers and non-completers

|  | **Completers (n=36)** | | **Non-completers (n=13)** | |
| --- | --- | --- | --- | --- |
|  | **n** | **Values**^a^ | **n** | **Values**^a^ |
| **Diagnoses**  IBS-D  IBS-M | 15  21 | 42  58 | 4  9 | 31  69 |
| **Demographics**  Age  Female  Social status  Single  Cohabitant/ married  Education, y  <13  13-17  >17 | 36  24  9  27  8  13  15 | 37 (11)  67  25  75  22  36  42 | 13  12  3  10  0  11  2 | 39 (13)  92  23  77  0  85  15 |

The table displays baseline characteristics the participants in the study who completed and did not complete the intervention. ^a^All values are given in percentages, except age which is given in mean (SD).

Supplementary Table 2: Percentage of participants scoring “more/ worse than usual” or “much more/worse than usual” on the CFQ-11 questions at baseline and follow up.

| **CFQ-11 question** | | **Baseline** | **Week 12** |
| --- | --- | --- | --- |
| 1 | Do you have problems with tiredness? | 72 | 28 |
| 2 | Do you need to rest more? | 61 | 31 |
| 3 | "Do you feel sleepy or drowsy?" | 75 | 31 |
| 4 | "Do you have problems starting things?" | 47 | 19 |
| 5 | "Do you lack energy?" | 61 | 31 |
| 6 | "Do you have less strength in your muscles?" | 39 | 11 |
| 7 | "Do you feel weak?" | 31 | 14 |
| 8 | "Do you have difficulties concentrating?" | 50 | 19 |
| 9 | "Do you make slips of the tongue when speaking?" | 17 | 8 |
| 10 | "Do you find it more difficult to find the right words?" | 44 | 22 |
| 11 | "How is you memory?" | 39 | 14 |

The table displays the percentage scoring “1” using the bimodal scoring schemes for the CFQ-11 on the individual questions. Abbreviations: CFQ-11, Chalder fatigue scale

Supplementary Table 3: Associations between change in core GI symptoms and change in symptoms of fatigue, anxiety, depression and attention from baseline to the 12 week follow up after the dietary intervention

|  | **Rho** | **p** |
| --- | --- | --- |
| **Fatigue (n=26)** | 0.02 | 0.909 |
| **Anxiety (n=34)** | 0.30 | 0.090 |
| **Depression (n=34)** | 0.26 | 0.134 |
| **Attention (n=29)** | |  |
| Detectability  Omissions  Commissions  Perseverations  HRT  HRT SD  Variability  HRT Block change  HRT ISI Change | 0.18  0.19  -0.05  0.16  0.24  0.10  -0.16  -0.04  0.28 | 0.362  0.329  0.795  0.413  0.215  0.598  0.412  0.834  0.148 |

Spearman correlations between change in IBS symptom severity and changes in fatigue, anxiety, depression, and attention variables from the CPT-3. Abbreviations: CPT-3, Continuous Performance Test-3; HRT, Hit reaction time; IBS; Irritable bowel syndrome; ISI, Inter-stimulus interval.
